# Is Thoracic Aortic Diameter an Independent Predictor of Cardiovascular Disease and Mortality? A Narrative Review

**DOI:** 10.3389/fcvm.2022.867026

**Published:** 2022-04-29

**Authors:** Marijana Tadic, Elisa Gherbesi, Carla Sala, Stefano Carugo, Cesare Cuspidi

**Affiliations:** ^1^Department of Cardiology, University Hospital “Dr. Dragisa Misovic-Dedinje”, Belgrade, Serbia; ^2^Department of Clinical Sciences and Community Health, Fondazione Ospedale Maggiore, IRCCS Policlinico di Milano, University of Milano, Milan, Italy; ^3^Department of Medicine and Surgery, University of Milano-Bicocca, Milan, Italy

**Keywords:** cardiovascular events, mortality, echocardiography, computed tomography, aortic diameter

## Abstract

Subclinical alterations in cardiac structure and function include a variety of abnormal phenotypes of recognized adverse prognostic values, such as left ventricular hypertrophy (LVH), concentric remodeling, systolic/diastolic dysfunction, left atrial dilatation, and alterations of LV geometry. The excess cardiovascular risk associated with these markers has been documented in multiple clinical settings, such as the general population, hypertensive cohorts, patients with coronary heart disease, diabetes mellitus, chronic heart failure, and chronic kidney disease. On the contrary, the value of aortic root (AR) and ascending aortic diameter in predicting cardiovascular outcomes and all-cause mortality in populations free from overt aortic pathology is still debated. The present review, aimed at pointing out the prognostic implications of thoracic aortic dimensions in populations free from known connective and aortic diseases, suggests that available evidence supporting an association between aortic diameter and cardiovascular events, and all-cause mortality is based on the limited number of studies, conducted with different imaging techniques and definition of the aortic phenotype.

## Introduction

Subclinical target organ damage (TOD) refers to the structural and functional alterations of the cardiovascular system associated with unhealthy risk factors, among which systemic hypertension stands out (1, 2). Asymptomatic alterations of the cardiovascular system reflect an intermediate step in the disease continuum linking hypertension and coexistent risk factors, such as dyslipidemia, obesity, and diabetes mellitus, to non-fatal and fatal cardiovascular events (3). A large body of evidence supports the view that subclinical TOD simultaneously occurs in the heart, brain, eye, kidney, and peripheral arteries presumably because cardiac and vascular tissues are similarly exposed to hemodynamic, neural, and hormonal stimuli operating in hypertension (4, 5).

Hypertensive heart disease represents one of the most important manifestations of subclinical TOD due to its high prevalence, possibly related to the early onset of this adverse phenotype in the natural history of hypertension and to its prognostic significance independent of traditional risk factors, including office blood pressure (BP) levels (6, 7). Furthermore, reversal of cardiac TOD, as assessed by echocardiography (i.e., left ventricular mass reduction), at the difference from other markers, such as carotid intima–media thickness and ankle–brachial index, has been consistently reported to be a reliable indicator of the protective effects of non-pharmacological and pharmacological antihypertensive therapy (8, 9). Echocardiographic left ventricular hypertrophy (LVH) is widely recognized as a key biomarker of hypertensive heart disease and a powerful predictor of cardiovascular morbidity and mortality in hypertensive patients as well as in different clinical settings, such as members of the general population, patients with coronary heart disease, diabetes mellitus, chronic heart failure (HF), and kidney disease (10–13). It should be underlined, however, that cardiac TOD, in addition to LVH, includes other important markers, namely LV geometry alterations, left atrial size, and aortic root (AR) dilatation as well as systolic/diastolic dysfunction that, alone or in association with LVH, may improve cardiovascular risk stratification (14). The independent role of concentric LV geometry, atrial dilatation, and systolic and diastolic dysfunction in predicting cardiovascular outcomes has been proven, with only some exceptions, by several studies carried out in patients with hypertension and in general population-based samples (15, 16). On the contrary, evidence on the prognostic value of AR and ascending aortic diameter in populations free from known aortic pathological conditions is very scanty. Consequently, the hypertension guidelines did not include aortic diameter among the markers of cardiac TOD useful for the evaluation of hypertensive heart disease (2). Therefore, this article is aimed at pointing out available evidence on the prognostic implications of thoracic aortic dimensions after excluding from the review specific clinical conditions, such as connective diseases (i.e., Marfan’s syndrome and Ehlers–Danlos syndrome) and aortic aneurysms.

## Methods

This article was prepared in accordance with the Narrative Review Checklist (available at http://dx.doi.org/10.21037/jtd-20-2728). The medical literature was reviewed to identify all articles evaluating the relationship between AR and aortic ascending diameter with incident cardiovascular events and mortality. A computerized search was performed using Pub-Med, OVID, EMBASE, and Cochrane library databases from inception up to December 31st 2021. Studies were identified by using the following search terms: “aortic root,” “ascending aorta,” “vascular damage,” “echocardiography,” “computed tomography,” “cardiovascular events,” “cardiovascular prognosis,” and “mortality.” Checks of the reference lists of selected papers and pertinent reviews complemented the electronic search. Data were examined and extracted by three independent investigators (EG, CC, and MT).

## Results

**FIGURE 1:**
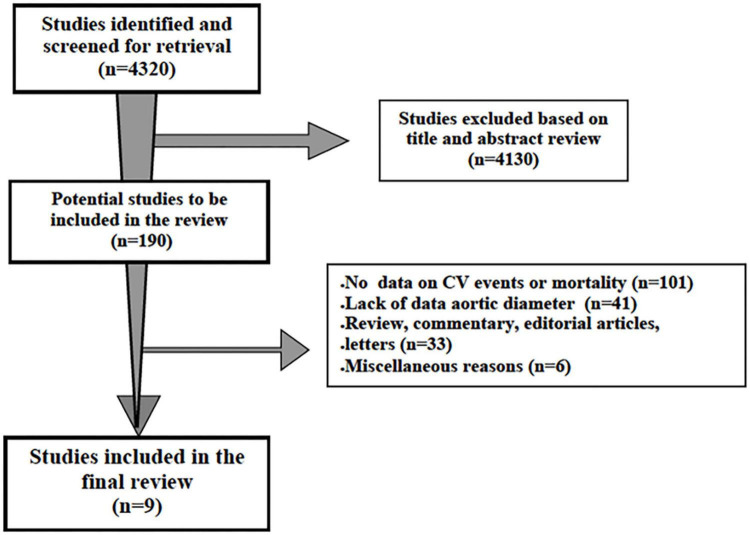
Schematic flowchart for the selection of studies.

The first literature search identified 4,320 papers. After the initial screening of titles and abstracts, 4,130 studies were excluded as they were not related to the topic. Therefore, 190 studies were reviewed; of these, 101 did not report data on incident non-fatal or fatal cardiovascular events or all-cause mortality and 41 on AR diameter or ascending aorta data, 33 were review, commentary, editorial articles, and 6 were excluded for miscellaneous reasons. A total of 9 studies, including participants without underlying known aortic pathologies (i.e., aneurysms) or connective diseases and containing sufficient clinical and cardiac imaging data, were included in the final review (17–25) (Figure 1). The Newcastle–Ottawa Score, used for assessing the quality of the studies, ranged from 7 to 9, and the mean score was 7.8. Therefore, no study was excluded based on its limited quality.

### Characteristics of the Studies

On the whole, 39,969 individuals were included in 9 studies (sample size ranging from 423 to 10,410 participants), performed in three continental areas (Europe = 4, North America = 4, Asia = 1).

Table 1 shows demographic and clinical characteristics of patients of selected studies, such as sample size, setting, mean age, prevalence of men, duration of follow-up, pre-specified outcomes of interest, and their association with baseline AR and ascending aortic diameter.

**TABLE 1:** Summary of longitudinal studies that addressed the relationship aortic root and ascending aortic diameter with cardiovascular prognosis and/or all-cause death.

References	Sample size (*n*)	Setting	Age (years)	Men%	Duration of FU	Outcomes	Main findings (and imaging tool)
Gardin et al. (17)	3,933	Elderly free of CVD	73 ± 6	42	10 years	Incident MI, CHF, Stroke, all-cause Mortality	Absolute ARD was predictive of incident CHF, stroke, CVD mortality, and all-cause mortality, but not of incident MI. (M-mode 2D guided Echocardiography).
Lai et al. (18)	1,851	General population	58 ± 10	44	12 years	Incident all-cause death	ARD indexed to BSA was predictive of all-cause death in participants < 65 years (M-mode 2D guided Echocardiography).
Gondrie et al. (19)	10,410	Population without history of CVD	63 (40–96)	60	17 months	Incident CV events	Ascending aortic diameter was associated to increased risk of CV events (Computed Tomography)
Lam et al. (20)	6,493	General population	56 ± 14	46	8 years	Incident HF	ARD was predictive of incident HF. (M-mode 2D guided Echocardiography)
Cuspidi et al. (21)	1,860	General population	50 ± 14	50	12 years	Incident CV events	ARD indexed to height were predictive of non-fatal and fatal CV events. (M-mode 2D guided Echocardiography)
Kamimura et al. (22)	3,108	Community-based black cohort	56 ± 12	31	8 years	Incident CV events, all-cause mortality	ARD, ARD indexed to BSA and height were predictive of non-fatal and fatal CV events and all-cause mortality (2D Echocardiography).
Qazi et al. (23)	3,318	General population	49 ± 10	51	9 years	Incident CV events	Ascending aortic diameter was not associated with excess of fatal and non-fatal CV events. (Computed Tomography).
Canciello et al. (24)	8,573	Hypertensive cohort	53 ± 12	58	4 years	Incident CV events	ARD indexed to height was independent predictor of CV events (2D Echocardiography).
Leone et al. (25)	423	Hypertensive cohort	53 ± 13	78	7 years	Incident CV events	Ascending aortic diameter was independent predictor of CV events. (2D Echocardiography).

*ARD, aortic root diameter; BSA, body surface area; CHF, chronic heart failure; CVD, cardiovascular disease; FU, follow-up; MI, myocardial infarction. Data are presented as absolute numbers, percentage, mean ± SD, Inter quartile range.*

The mean age range was 49–73 years (17, 23); 51% of participants were men. The majority of studies included free-living members of the general population, two studies were carried out in hypertensive cohorts (24, 25) and one study in patients undergoing chest computed tomography (CT) for non-cardiovascular indications (19). The duration of the follow-up period ranged from 17 months (19) to 12 years (18, 21).

**FIGURE 2:**
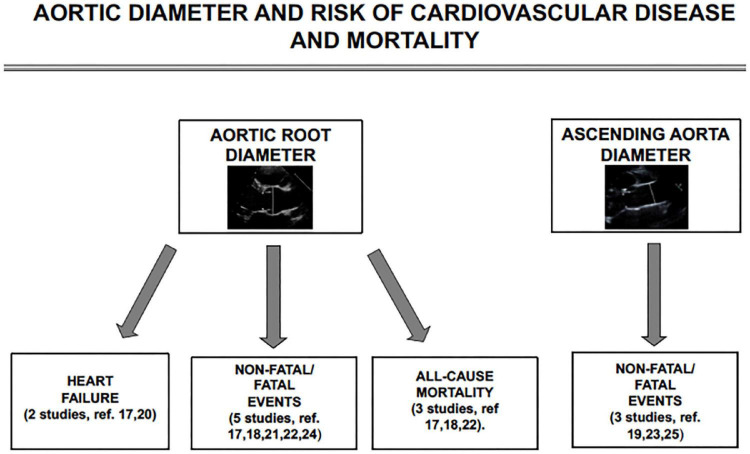
Schematic flow-chart for studies targeting the association of aortic root and ascending aortic diameter with incident heart failure, non-fatal cardiovascular events, and all-cause mortality.

Figure 2 provides a flow-chart targeting, the association of AR and ascending aortic diameter with the outcomes of interest in the 9 studies included in the review.

### Imaging Procedures and Main Findings

Two different cardiac imaging methods were employed in the studies included in the present review: echocardiography (*n* = 7) and CT (*n* = 2). Echocardiographic studies were performed according to recommendations of major contemporary guidelines. AR diameter was measured in the parasternal long-axis view at the level of Valsalva’s sinuses in six echocardiographic studies (17, 18, 20–22, 24). The diameter of ascending aorta was the vascular phenotype of interest in both CT studies (19, 23) and in one of the echocardiographic studies (25).

### All-Cause Mortality

Three of the nine studies that analyzed the relationship between aortic diameter and all-cause mortality (17, 18, 22) found that the baseline values of absolute AR diameter (17) and indexed diameter (18, 22) were independent predictors of this fatal outcome. It is worth noting, however, that in the study by Lai et al. (18), this association persisted, after the adjustment for confounders, only in participants = 65 years of age.

### Non-fatal and Fatal Cardiovascular Events

Two out of three studies (21, 24) found that AR diameter indexed to BSA or height predicted non-fatal and fatal cardiovascular events, regardless of traditional risk factors. This was not the case, however, in the PAMELA study in which the association of AR diameter with incident cardiovascular events lost its significance when LV mass index was included in the multivariate analysis (21). Studies targeting ascending aortic diameter (19, 23, 25) showed non-univocal findings. Among the participants from the Framingham offspring and third-generation cohorts, the enlarged ascending aorta was not significantly associated with cardiovascular events (23). In one of the two studies documenting an excess risk of cardiovascular events related to the enlargement of ascending aorta diameter, the adjustment for confounding factors was limited to age and sex.

### Heart Failure

Among the individuals belonging to Cardiovascular Health Study (CHS), an increased AR diameter was found to be an independent predictor of incident HF in men after the adjustment for several confounders, including electrocardiographic LV mass (17). The Framingham Heart Study researchers reported a positive association between AR diameter with incident chronic HF; this relationship, however, lost statistical significance after the adjustment for echocardiographic LV mass in addition to clinical risk factors (20).

## Discussion

The progressive arterial remodeling related to age represents a key mechanism in the pathogenesis of cardiovascular disease (26, 27). For many decades, numerous imaging-based and post-mortem studies have shown an association between the aging process and dilatation of the thoracic and abdominal aorta (28, 29). Age-related dilation of the aorta due to long-term exposure to cardiovascular risk factors, such as hypertension, metabolic disorders, sleep apnea syndrome, and smoking, has been related to structural changes in the aortic wall, such as calcification, collagen deposition, elastin fractures, and reduced elastin content (30). Although elevated BP levels tend to increase aortic wall stress, the contribution of BP to aortic dilatation appears to be substantially lower compared to other factors, such as age, gender, and body size measures (31). This view has been supported by cross-sectional studies targeting AR dilatation and carried out in the general population and in the hypertensive setting. Prevalence rates of AR dilatation in the PAMELA population (*n* = 1,860) varied from 5.6% (AR/BSA) to 9.6% (AR/height), the men/women ratio being approximately 1.1 with both criteria (21). A meta-analysis of eight studies, including a pooled population of 10,791 hypertensive patients, documented that the overall prevalence of AR dilatation was 9.1%, quite similar to the PAMELA study (32).

It should be noted, however, that available evidence regarding dynamic changes over time in AR diameter in the community and in hypertensive cohorts suggests somewhat different conclusions from cross-sectional studies. Indeed, among the participants to the PAMELA study, the incidence of new AR dilatation over the 10-year follow-up period ranged from 3.4% (AR/BSA) to 4.4% (AR/height) (33). In difference, the Campania Salute Network study, including 4,856 hypertensive patients, showed that as many as 366 participants (11%) with normal AR diameter at baseline developed AR dilatation during a follow-up of 6 years (34).

In understanding the prognostic role of aortic diameter, the following important questions need to be carefully considered: (I) is its predictive meaning independent of traditional risk factors and, more importantly, of other parameters of LV structure and function, namely LV mass index?; (II) do sex and age influence the relationship between aortic diameter and outcomes?; (III) is aortic dilatation an independent correlate of both cardiovascular and non-cardiovascular mortality?

Regarding the first question, eight of the nine studies that found a positive relationship between aortic diameter and cardiovascular outcomes provided statistical data adjusted for several key confounders; only in one study the adjustment was limited to age and gender (23). It is worth noting, however, that inclusion of LV mass in statistical models abolished the prognostic significance of aortic diameter in predicting HF (20), non-fatal and fatal cardiovascular events (21), and all-cause mortality in hypertensive patients on anti-hypertensive medications (17). In contrast, two Italian studies carried out in patients referred to specialist hypertension centers showed that AR and ascending aorta diameter were independent predictors of cardiovascular events regardless of LVH and other common confounders (24, 25).

Only a few studies performed subgroup analyses stratified by gender (17, 18) and age (18). The CHS, based on a bi-racial sample of the general population, including 3,993 elderly without overt cardiovascular disease, reported that an enlarged AR diameter was associated with a greater risk for incident HF in men (*HR*: 1.47; *p* = 0.014), but not in women (17). No gender differences were found for other outcomes, such as stroke, cardiovascular mortality, and all-cause mortality.

In the Chin–Shan Community Cardiovascular Cohort, the association between AR dilatation and non-cardiovascular death was found in adults < 65 years, but not in older participants, without differences in the analysis stratified by sex (18).

Evidence on the adverse impact of aortic dilation on all-cause mortality is based on two general population-based samples (18, 22) and on a subgroup of treated hypertensive elderly people belonging to CHS (17). As previously mentioned, however, it should be underlined that adjustment for LV mass abolished the significance of this relationship in the CHS cohort (17) and that the Jackson Heart Study did not include this echocardiographic parameter among the confounding factors (22).

As for cardiovascular events, no specific evidence is available about the predictive role of aortic dilatation on cardiovascular mortality. In fact, most studies examined exclusively a composite of non-fatal and fatal stroke, coronary events, and HF requiring hospitalization (18, 19, 21–25). Additional events, such as transient ischemic attacks, atrial fibrillation, cardioverter-defibrillator implants, and surgery involving major aorta branches, have been included in the composite outcome in some studies, but not in others. The CHS, the only study providing separate data on cardiac and cerebrovascular outcomes, showed that aortic dilatation predicted an increased risk for stroke and HF (in men) but not for myocardial infarction (17). As for HF, the Framingham Heart Study focused on this specific outcome showed that participants with a greater AR diameter experienced a higher risk of incident HF over an 8-year period of follow-up (18). As the association of AR diameter with incident HF was rendered non-significant after the adjustment for LV mass, a possible interpretation of the link between aortic dilation and HF is that LV mass mediates the progression to HF in presence of AR remodeling. In this regard, numerous studies have shown an independent association between LVH and aortic dilatation assessed with different imaging techniques, in several clinical settings, such as hypertensive patients, elderly individuals, and patients with aortic aneurysms (35–38). These observations suggest that alterations of the aortic wall structure/function associated with dilatation may contribute to increased LV afterload, an important factor leading to LVH. Thus, the dilatation of the most proximal arterial segment (i.e., AR and ascending tract), in addition to being a known risk factor for aortic dissection, can be considered a sign of TOD paralleling other cardiac markers of established prognostic value (39, 40). In particular, the association between aortic dilatation and LVH emphasizes the role of combined arterial–ventricular remodeling in the progression of cardiovascular continuum. Findings from the general population and hypertensive cohorts showed that the incidence of cardiovascular events was significantly increased when changes in LV structure were paralleled by those in aortic dimension and the fully adjusted risk of cardiovascular events was markedly greater in individuals with LVH and aortic dilatation than in their counterparts with LVH alone (21, 24).

It should be remarked that the mechanisms underlying aortic dilation are extremely complex and related to the interplay of adverse hemodynamic and non-hemodynamic factors, such as wall stress, inflammatory processes, altered regulation of growth factors, activation of the sympathetic nervous system, and imbalance between proteases and corresponding inhibitors resulting in degradation and fragmentation of extracellular matrix (41–44). The pathophysiological mechanisms linking aortic dilation to cardiovascular events must be considered mostly hypothetical. There are several pathophysiological mechanisms in the relationship between aortic dilatation and CV events that may be considered. Tissue remodeling of the aortic wall (i.e., reduction in elastin fiber, and increased collagen and calcium deposition) resulting in increased arterial stiffness may contribute to the relationship between aortic diameter and cardiovascular events. Furthermore, it has been speculated that combined aortic and ventricular remodeling have a pivotal role in the pathogenesis of HF. However, proximal aortic dilation may be considered as a marker of the impact of multiple cardiovascular risk factors of established prognostic significance rather than a mediator of cardiac, cerebrovascular events, and all-cause mortality.

In conclusion, current evidence supporting an association between aortic diameter and cardiovascular events as well as all-cause mortality in populations without overt vascular pathology is based on the limited number of studies, conducted in different settings (i.e., elderly individuals, free-living members of the general population, and hypertensive cohorts) with different imaging techniques (i.e., echocardiography and CT), based on different definitions of the aortic phenotype of interest (i.e., AR diameter or ascending aorta diameter), a wide range of follow-up duration (i.e., 12–144 months) and heterogeneous primary outcomes (i.e., cardiovascular events, HF, and total mortality) (45). Furthermore, an accumulating amount of evidence suggests that a single measurement of aortic diameter, is an unreliable indicator of vascular damage of prognostic significance, especially when this parameter is not indexed by body size (17, 19, 23, 24, 46). Therefore, further studies using more homogeneous methods, populations, and outcomes are still needed.

## Author Contributions

MT: writing and reviewing. EG and CS: methodology and statistics. SC: searching the literature. CC: writing, methodology, statics, reviewing, and revising. All authors contributed to the article and approved the submitted version.

## Conflict of Interest

The authors declare that the research was conducted in the absence of any commercial or financial relationships that could be construed as a potential conflict of interest. The reviewer RF declared a shared affiliation with one of the author CC to the handling editor at the time of review.

## Publisher’s Note

All claims expressed in this article are solely those of the authors and do not necessarily represent those of their affiliated organizations, or those of the publisher, the editors and the reviewers. Any product that may be evaluated in this article, or claim that may be made by its manufacturer, is not guaranteed or endorsed by the publisher.
